# Quantification of atrial dynamics using cardiovascular magnetic resonance: inter-study reproducibility

**DOI:** 10.1186/s12968-015-0140-2

**Published:** 2015-05-17

**Authors:** Johannes T. Kowallick, Geraint Morton, Pablo Lamata, Roy Jogiya, Shelby Kutty, Gerd Hasenfuß, Joachim Lotz, Eike Nagel, Amedeo Chiribiri, Andreas Schuster

**Affiliations:** Division of Imaging Sciences and Biomedical Engineering, The Rayne Institute, St Thomas’ Hospital, King’s College London, London, UK; Institute for Diagnostic and Interventional Radiology, Georg-August-University Göttingen, Göttingen, Germany; Portsmouth Hospitals NHS Trust, Portsmouth, UK; Children’s Hospital and Medical Center, University of Nebraska College of Medicine, Omaha, NE USA; Department of Computer Science, University of Oxford, Oxford, UK; Department of Cardiology and Pneumology, Georg-August-University Göttingen, Göttingen, Germany; DZHK (German Centre for Cardiovascular Research), partner site Göttingen, Göttingen, Germany; Division of Cardiovascular Imaging, Goethe University Frankfurt and German Centre for Cardiovascular Research (DZHK, partner site Rhine-Main), Frankfurt, Germany

**Keywords:** Inter-study reproducibility, Atrial function, Cardiovascular magnetic resonance, Volumetric indexes, Feature tracking, Reservoir function, Conduit function, Contractile function

## Abstract

**Background:**

Cardiovascular magnetic resonance (CMR) offers quantification of phasic atrial functions based on volumetric assessment and more recently, on CMR feature tracking (CMR-FT) quantitative strain and strain rate (SR) deformation imaging. Inter-study reproducibility is a key requirement for longitudinal studies but has not been defined for CMR-based quantification of left atrial (LA) and right atrial (RA) dynamics.

**Methods:**

Long-axis 2- and 4-chamber cine images were acquired at 9:00 (Exam A), 9:30 (Exam B) and 14:00 (Exam C) in 16 healthy volunteers. LA and RA reservoir, conduit and contractile booster pump functions were quantified by volumetric indexes as derived from fractional volume changes and by strain and SR as derived from CMR-FT. Exam A and B were compared to assess the inter-study reproducibility. Morning and afternoon scans were compared to address possible diurnal variation of atrial function.

**Results:**

Inter-study reproducibility was within acceptable limits for all LA and RA volumetric, strain and SR parameters. Inter-study reproducibility was better for volumetric indexes and strain than for SR parameters and better for LA than for RA dynamics. For the LA, reservoir function showed the best reproducibility (intraclass correlation coefficient (ICC) 0.94–0.97, coefficient of variation (CoV) 4.5–8.2 %), followed by conduit (ICC 0.78–0.97, CoV 8.2–18.5 %) and booster pump function (ICC 0.71–0.95, CoV 18.3–22.7). Similarly, for the RA, reproducibility was best for reservoir function (ICC 0.76–0.96, CoV 7.5–24.0 %) followed by conduit (ICC 0.67–0.91, CoV 13.9–35.9) and booster pump function (ICC 0.73–0.90, CoV 19.4–32.3). Atrial dynamics were not measurably affected by diurnal variation between morning and afternoon scans.

**Conclusions:**

Inter-study reproducibility for CMR-based derivation of LA and RA functions is acceptable using either volumetric, strain or SR parameters with LA function showing higher reproducibility than RA function assessment. Amongst the different functional components, reservoir function is most reproducibly assessed by either technique followed by conduit and booster pump function, which needs to be considered in future longitudinal research studies.

## Background

Heart failure of different origins remains a major determinant of mortality [1]. Pathophysiological changes involve impaired left and right ventricular systolic and diastolic function as well as the affection of atrial mechanics including size and function [2]. There is evidence to suggest that impaired left atrial (LA) performance after acute myocardial infarction is associated with adverse outcome [3]. Speckle tracking echocardiography and more recently cardiovascular magnetic resonance (CMR) myocardial feature tracking (CMR-FT) provide accurate quantification of the three basic aspects of atrial physiology [2, 4–7]: 1.) Reservoir function (collection of venous return during ventricular systole), 2.) conduit function (passage of blood to the ventricles during early ventricular diastole) and 3.) contractile booster pump function (active augmentation of ventricular filling during late ventricular diastole).

Generally when using CMR, atrial physiology may be quantified using volumetric indexes as derived from fractional volume changes [4, 6, 7] or CMR-FT based analysis of standard steady-state free precession (SSFP) images [8]. Impaired LA function detected with CMR-FT accurately identifies patients with heart failure and preserved ejection fraction and hypertrophic cardiomyopathy [8], shows close correlation with LV fibrosis [9] and represents a powerful prognostic marker for the development of heart failure in the general population [10]. Especially for the latter indication and serial longitudinal follow-up scans inter-study reproducibility is a key requirement. However, inter-study reproducibility has neither been reported for volumetric indexes nor CMR-FT derived atrial function assessment. Consequently, the aim of the present study was to investigate the inter-study reproducibility of CMR derived LA and right atrial (RA) function assessment as determined by phasic volumetric analysis as well as by CMR-FT derived atrial strain and SR.

## Methods

The St Thomas’ Hospital Research Ethics Committee approved the study. The study complies with the Declaration of Helsinki and its later amendments. 16 healthy participants were included. All participants gave written informed consent before the CMR measurements. Exclusion criteria included known cardiac, respiratory or renal disease or an absolute contraindication to CMR.

### CMR imaging

Participants underwent 3 CMR examinations on the same day. All imaging was performed at 3 Tesla (Achieva, Philips Medical Systems, Best, The Netherlands) with participants in the supine position using a 32-channel phased array receiver cardiac coil. On the study day participants were encouraged to fast from midnight. The first CMR examination was performed at 9:00 (Exam A), immediately followed by a second exam at 9:30 (Exam B). In order to try and maximise physiological changes, participants then left the department to eat and drink as normal. They returned at 14:00 for the third scan (Exam C). Exams A and B were acquired to assess for the inherent variability of CMR-FT quantification of atrial dynamics. Exams A and C were used for the assessment of potential diurnal physiological alterations due to circadian rhythms or different states of hydration.

The CMR protocol included an initial survey, a coil reference scan and planning to define imaging planes, independently for all three CMR scans. Cine images were acquired using a standard ECG-gated balanced steady state free precession (SSFP) sequence in long-axis 2- and 4-chamber views (in-plane resolution 1.8 × 2 mm, slice thickness 8 mm, 30 time frames). The protocol was identically repeated for all three scans and for all volunteers. Participants were removed from the scanner between different exams.

### Volumetric analysis

**Fig. 1:**
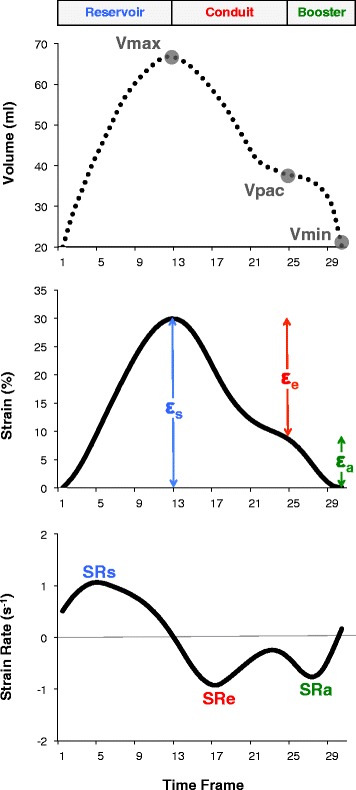
Quantification of atrial function as derived from volumetric analysis and CMR-FT derived strain and strain rate (SR) parameters. Atrial analysis comprised reservoir, conduit and booster pump function. For the calculation of fractional volume changes, atrial volumes were measured in three time frames according to the maximum (Vmax), minimum (Vmin) and atrial volume prior to atrial contraction (Vpac) resulting in ‘EF Total’ corresponding to reservoir function and global atrial function, ‘EF Passive’ corresponding to conduit function and ‘EF Booster’ corresponding to atrial contractile booster pump function (please see equations in the methods section under volumetric analysis). Total strain (ε_s_) and peak positive strain rate (SRs) correspond to reservoir function. Passive strain (ε_e_) and peak early negative strain rate (SRe) correspond to conduit function. Active strain (ε_a_) and peak late negative strain rate (SRa) correspond to contractile booster pump function

Volumetric analysis was performed with commercially available software (CMR 42, Circle, Canada) in a random order by a blinded experienced observer. Semi-automated tracking of the LA area and length were performed in the 2- and 4-chamber views excluding pulmonary veins and the LA appendage. RA area and length were tracked in the 4-chamber view, only. LA volumes and RA volumes were calculated according to the biplane area-length and the single plane area-length method, respectively [11, 12]. Maximum LA and RA volumes were assessed at ventricular end-systole (Vmax), at ventricular diastole prior to atrial contraction (Vpac) and at late ventricular diastole after atrial contraction (Vmin) (Fig. 1) [4, 6, 7]. Left and right total atrial emptying fraction (EF Total, corresponding to atrial reservoir function and atrial global function), passive atrial emptying fraction (EF Passive, corresponding to atrial conduit function) and active atrial emptying fraction (EF Booster, corresponding to atrial contractile booster pump function) were defined as fractional volume changes according to the following equations for both atria, respectively [4, 6, 7]:

$$ EF\  Total = \frac{\left( Vmax- Vmin\right)*100\kern0.5em }{V\  max} $$

$$ EF\  Passive = \frac{\left( Vmax- Vpac\right)\ *100\ }{Vmax} $$

$$ EF\  Booster = \frac{\left( Vpac- Vmin\right)*100}{Vpac} $$

### Feature tracking

CMR-FT was performed using dedicated software (TomTec Imaging Systems, 2 days CPA MR, Cardiac Performance Analysis, Version 1.1.2, Unterschleissheim, Germany) as previously described [8]. Images were analysed in a random order by a blinded experienced observer. LA endocardial borders were tracked in the 2- and 4-chamber views. RA endocardial borders were tracked in the 4-chamber view. Tracking was repeated for three times in each view. Results were based on the average of 6 segments in each view and the three repeated measurements. In the presence of insufficient tracking quality (e.g. due to the presence of pulmonary veins) the corresponding segment was excluded from the analysis. CMR-FT was performed with the reference point set after atrial contraction (“QRS-QRS gating” [2]). Three aspects of LA and RA strain were analysed (Fig. 1) [8, 13, 14]: passive strain (ε_e_, corresponding to atrial conduit function), active strain (ε_a,_ corresponding to atrial contractile booster pump function) and total strain, the sum of passive and active strain (ε_s,_ corresponding to atrial reservoir function). Accordingly, three SR parameters were evaluated (Fig. 1): peak positive strain rate (SRs, corresponding to atrial reservoir function), peak early negative strain rate (SRe, corresponding to atrial conduit function) and peak late negative strain rate (SRa, corresponding to atrial contractile booster pump function) [2, 8].

### Statistical analysis

Statistical analysis was performed using Microsoft Excel and IBM SPSS Statistics version 22 for Macintosh. Data from the repeated exams are expressed as mean ± standard deviation. The Shapiro-Wilk test was applied to test for normally distributed data. Since LA EF Passive, LA Vmin and RA EF Conduit were not normally distributed, a natural logarithmic transformation was performed. The Shapiro-Wilk test was reapplied to check for normal distribution after natural logarithmic transformation. A one-way analysis of variance (ANOVA) for repeated measures with Bonferroni post hoc adjustment in case of significance was conducted to evaluate the null hypothesis that there is no change in atrial functional elements between the repeated Exams A, B and C. All p values < 0.05 were considered statistically significant.

The inter-study variability was assessed by intraclass correlation coefficients (ICC) using a model of absolute agreement. Agreement was considered excellent when ICC > 0.74, good when ICC = 0.60–0.74, fair when ICC = 0.40–0.59, and poor when ICC < 0.4 [15]. The mean difference with 95 % limits of agreement (±2 standard deviations) between the repeated measurements was calculated according to method of Bland and Altman [16]. Coefficients of variation (CoV), defined as the standard deviation of the differences divided by the mean [17], were calculated. Furthermore, study sample sizes required to detect a relative 5 %, 10 %, 15 % and 20 % change in atrial functional parameters with a power of 90 % and an α error of 0.05 were calculated as follows [17]:

$$ \boldsymbol{n}=\boldsymbol{f}\ \left(\boldsymbol{\alpha},\ \boldsymbol{P}\right){\boldsymbol{\sigma}}^{\boldsymbol{2}}\frac{\boldsymbol{2}}{{\boldsymbol{\delta}}^{\boldsymbol{2}}} $$

where n is the sample size, f = 10.5 for α 0.05 and P 0.9, σ the inter-study standard deviation and δ the magnitude of the differences to be detected.

## Results

Sixteen healthy volunteers (8 male, 8 female) aged 27.9 ± 5.7 with a body mass index of 26.2 ± 6.8 kg/m^2^ were included in the study. One participant did not attend Exam C. In one measurement (Exam C) there was no detectable lumen after LA contraction in the 4-chamber view, so that neither LA volumetric nor CMR-FT analysis could be performed. In one scan (Exam C) it was not possible to perform CMR-FT of the RA due to severe flow artefacts. Consequently, only the volumetric analysis was included in the study. In total 16 cases were compared to assess the inter-study reproducibility for LA and RA volumetric and CMR-FT derived function (Exam A vs. Exam B). 14 cases (LA volumetric analysis, LA and RA CMR-FT) and 15 cases (RA volumetric analysis) were compared for the assessment of diurnal variation (Exam A/B vs. Exam C), respectively. 94. and 87.7 % of all segments could be included in LA and RA CMR-FT analysis, respectively.

### Atrial dynamics

**Fig. 2:**
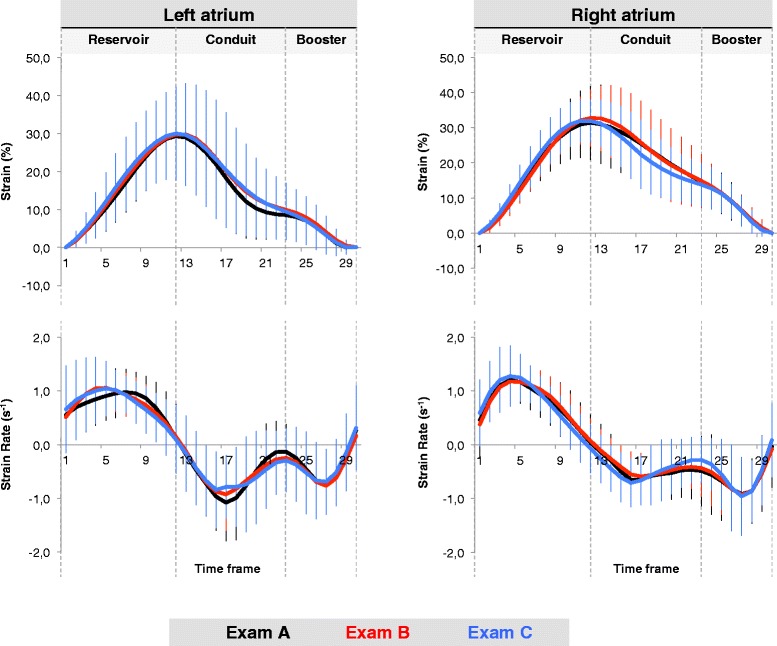
Atrial strain and strain rate profiles. Left and right atrial strain and strain rate profiles are displayed for all three Exams A-C. The three curves displayed for each parameter represent an average of the whole study group for the first, the second and the third CMR examination (Exams A-C) respectively (please see colour codes)

LA and RA volumes and volumetric functional indexes as well as CMR-FT derived strain and SR parameters are summarised in Table 1. Strain and SR profiles of all repetitions are displayed in Fig. 2. LA minimum volume was significantly different between the Exams A and B (p = 0.03). There were no significant differences in any LA or RA functional parameter between all three repeated exams. There was no measurable affection by diurnal variation of LA or RA functional elements.

**Table 1:** Right and left atrial dynamics

		**Left atrium**				**Right atrium**			
		**Exam A**	**Exam B**	**Exam C**	**P value** ^**a**^	**Exam A**	**Exam B**	**Exam C**	**P value** ^**a**^
**Atrial function**	**Volumetric index (%)**								
Reservoir; global	EF Total	66.3 ± 7.5	64.8 ± 5.8	68.8 ± 8.4	0.09	55.0 ± 9.6	54.5 ± 9.6	54.4 ± 6.3	0.68
Conduit	EF Passive	45.8 ± 7.5	45.2 ± 7.7	46.7 ± 9.8	0.62	34.2 ± 8.6	32.2 ± 8.3	30.9 ± 5.6	0.16
Booster pump	EF Booster	38.3 ± 7.5	35.6 ± 7.9	41.9 ± 7.6	0.40	32.4 ± 8.5	33.3 ± 9.1	34.0 ± 7.6	0.26
	**Strain (%)**								
Reservoir	ε_s_	31.8 ± 5.4	31.5 ± 5.3	31.3 ± 4.8	0.16	34.0 ± 9.5	34.6 ± 7.9	33.9 ± 6.7	0.41
Conduit	ε_e_	24.2 ± 5.6	23.7 ± 5.2	23.3 ± 4.9	0.16	22.2 ± 6.8	22.8 ± 5.3	22.6 ± 4.4	0.78
Booster pump	ε_a_	7.6 ± 3.1	7.7 ± 3.0	7.9 ± 2.7	0.77	11.8 ± 4.4	11.8 ± 4.4	11.3 ± 4.0	0.40
	**Strain Rate (s** ^**−1**^ **)**								
Reservoir	SR_s_	1.2 ± 0.2	1.2 ± 0.3	1.3 ± 0.3	0.91	1.3 ± 0.4	1.3 ± 0.3	1.4 ± 0.5	0.67
Conduit	SR_e_	−1.6 ± 0.4	−1.4 ± 0.3	−1.4 ± 0.3	0.06	−0.9 ± 0.3	−0.9 ± 0.3	−1.1 ± 0.4	0.18
Booster pump	SR_a_	−0.9 ± 0.4	−0.9 ± 0.4	−1.0 ± 0.4	0.56	−1.2 ± 0.7	−1.1 ± 0.6	−1.2 ± 0.7	0.98
	**Atrial volume (ml)**								
	Maximum	66.0 ± 16.2	67.0 ± 15.4	64.9 ± 17.8	0.55	80.0 ± 23.1	80.9 ± 24.3	78.7 ± 23.1	0.79
	Minimum	22.6 ± 8.6	23.8 ± 7.6	21.2 ± 11.1	0.03^b^	37.1 ± 15.5	37.9 ± 16.3	36.5 ± 13.2	0.68
	Prior to atrial contraction	36.1 ± 11.4	36.9 ± 10.7	35.8 ± 15.8	0.46	53.7 ± 19.8	55.6 ± 21.2	54.4 ± 17.0	0.42

Comparison of left and right atrial functional parameters between the repeated measurements. Values are given as mean ± standard deviation.

^a ^as derived from one-way ANOVA for repeated measures across the Exams A, B and C.

^b^ Significance between Exam A and Exam B (p = 0.03), as derived from one-way ANOVA for repeated measures with Bonferroni post hoc testing.

ε, strain; SR, strain rate; EF, emptying fraction.

### Inter-study reproducibility

Inter-study reproducibility was within acceptable limits for all LA and RA volumetric, strain and SR parameters. Bland-Altman analysis (mean differences ± 2SD), CoV and ICC for LA and RA functional parameters are summarised in Table 2. With respect to LA and RA functional elements, reproducibility was best for reservoir function, followed by conduit and booster pump function. With respect to the different methodologies, LA reproducibility was best for strain, followed by volumetric indexes and SR, whilst RA reproducibility was best for volumetric indexes, followed by strain and SR. LA strain and SR parameters derived from 2 chamber views had higher reproducibility than those derived from 4 chamber views (Table 3).

**Table 2:** Inter-study reproducibility

		**Left atrium**			**Right atrium**		
		**Mean difference ± 2 SD**	**CoV (%)**	**ICC (95 % CI)**	**Mean difference ± 2 SD**	**CoV (%)**	**ICC (95 % CI)**
**Atrial function**	**Volumetric index (%)**						
Reservoir; global	EF Total	1.5 ± 5.8	4.5	0.94 (0.81-0.98)	0.5 ± 8.0	7.5	0.96 (0.87-0.98)
Conduit	EF Passive	0.6 ± 11.3	12.6	0.84 (0.54-0.94)	1.9 ± 9.0	13.9	0.91 (0.75-0.97)
Booster pump	EF Booster	0.8 ± 8.5	21.6	0.71 (0.19-0.90)	1.4 ± 8.2	19.4	0.73 (0.26-0.90)
	**Strain (%)**						
Reservoir	ε_s_	0.3 ± 3.5	5.7	0.97 (0.92-0.99)	0.5 ± 11.1	16.6	0.89 (0.68-0.96)
Conduit	ε_e_	0.5 ± 3.9	8.2	0.97 (0.90-0.99)	0.6 ± 9.1	20.7	0.84 (0.53-0.94)
Booster pump	ε_a_	0.2 ± 2.7	18.3	0.95 (0.85-0.98)	0.1 ± 5.6	24.3	0.89 (0.68-0.96)
	**Strain Rate (s** ^**−1**^ **)**						
Reservoir	SR_s_	0.0 ± 0.2	8.2	0.96 (0.87-0.99)	0.0 ± 0.6	24.0	0.76 (0.29-0.92)
Conduit	SR_e_	0.2 ± 0.5	18.5	0.78 (0.36-0.93)	0.1 ± 0.6	35.9	0.67 (0.05-0.89)
Booster pump	SR_a_	0.0 ± 0.4	22.7	0.91 (0.75-0.97)	0.1 ± 0.7	32.2	0.90 (0.73-0.97)
	**Atrial volume (ml)**						
	Maximum	1.0 ± 8.8	6.8	0.98 (0.94-0.99)	0.9 ± 19.5	12.4	0.96 (0.88-0.99)
	Minimum	1.2 ± 4.6	10.0	0.98 (0.92-0.99)	0.8 ± 10.1	13.7	0.97 (0.93-0.99)
	Prior to atrial contraction	0.8 ± 7.3	10.3	0.97 (0.92-0.99)	1.9 ± 14.2	13.2	0.97 (0.91-0.99)

Inter-study reproducibility for left and right atrial functional parameters and volumes as determined by Bland-Altman analysis (mean difference ± 2SD), coefficients of variation (CoV) and intra-class correlation coefficients (ICC).

SD, standard deviation; CoV, coefficient of variation; ICC, intraclass correlation coefficient; CI, confidence interval.

**Table 3:** Inter-study reproducibility for CMR-FT derived LA dynamics in 2- and 4-chamber views

		**Left atrium (2-chamber view)**			**Left atrium (4-chamber view)**		
		**Mean difference ± 2 SD**	**CoV (%)**	**ICC (95 % CI)**	**Mean difference ± 2 SD**	**CoV (%)**	**ICC (95 % CI)**
**Atrial function**	**Strain (%)**						
Reservoir	ε_s_	0.7 ± 7.1	9.2	0.96 (0.89-0.99)	0.3 ± 8.2	17.4	0.84 (0.55-0.95)
Conduit	ε_e_	0.2 ± 6.3	10.9	0.97 (0.92-0.99)	0.3 ± 7.9	21.7	0.82 (0.48-0.94)
Booster pump	ε_a_	0.5 ± 2.2	22.2	0.92 (0.76-0.97)	0.0 ± 3.3	31.4	0.93 (0.80-0.98)
	**Strain Rate (s** ^**−1**^ **)**						
Reservoir	SR_s_	0.0 ± 0.3	9.9	0.96 (0.90-0.99)	0.0 ± 0.4	21.1	0.89 (0.67-0.96)
Conduit	SR_e_	0.0 ± 0.5	16.6	0.93 (0.80-0.98)	0.3 ± 0.9	32.6	0.45 (0.00-0.80)
Booster pump	SR_a_	0.1 ± 0.1	24.4	0.91 (0.73-0.97)	0.0 ± 0.5	37.4	0.76 (0.28-0.92)

Inter-study reproducibility for left atrial strain and strain rate parameters in 2- and 4-chamber views as determined by Bland-Altman analysis (mean difference ± 2SD), coefficients of variation (CoV) and intra-class correlation coefficients (ICC).

SD, standard deviation; CoV, coefficient of variation; ICC, intraclass correlation coefficient; CI, confidence interval.

Reproducibility was excellent for LA reservoir function (EF Total: ICC 0.94, CoV 4.5 %; ε_s_: ICC 0.97, CoV 5.7 %; SRs: ICC 0.96, CoV 8.2 %). Variability was higher for LA conduit function (EF Passive: ICC 0.84, CoV 12.6 %; ε_e_: ICC 0.97, CoV 8.2 %; SRe: ICC 0.78, CoV 18.5 %) and LA booster pump function (EF Booster: ICC 0.71, CoV 21.6 %; ε_a_: ICC 0.95, CoV 18.3 %; SRa: ICC 0.91, CoV 22.7 %).

Reproducibility was good for RA reservoir function (EF Total: ICC 0.96, CoV 7.5 %; ε_s_: ICC 0.89, CoV 16.6 %; SRs: ICC 0.76, CoV 24.0 %). Variability was higher for RA conduit function (EF Passive: ICC 0.91, CoV 13.9 %; ε_e_: ICC 0.84, CoV 20.7 %; SRe: ICC 0.67, CoV 35.9 %) and RA booster pump function (EF Booster: ICC 0.73, CoV 19.4 %; ε_a_: ICC 0.89, CoV 24.3 %; SRa: ICC 0.90, CoV 32.2 %).

### Sample size calculations

The differences in reproducibility between LA and RA atrial functional parameters are reflected in the sample size calculations. Sample sizes required to detect a relative 5, 10, 15 and 20 % change in atrial volumetric, strain or SR parameters are summarised in Table 4. Required sample sizes increase with smaller differences to be detected. Sample sizes are ranging between n = 2 to detect a relative 20 % change in LA reservoir function as determined by either strain or volumetric analysis (corresponding to a change in LA ε_s_ of 6.3 % and LA EF Total of 13.1 % in the present study) and n = 1085 to detect a 5 % change in RA conduit function as determined by SR (corresponding to a magnitude of RA SRe of 0.05 s^−1^ in the present study).

**Table 4:** Sample sizes

		**Left atrium**				**Right atrium**			
		**5 %**	**10 %**	**15 %**	**20 %**	**5 %**	**10 %**	**15 %**	**20 %**
**Atrial function**	**Volumetric index (%)**								
Reservoir; global	EF Total	18	5	2	2	47	12	6	3
Conduit	EF Passive	135	34	15	9	162	41	18	11
Booster pump	EF Booster	391	98	44	25	317	80	36	20
	**Strain (%)**								
Reservoir	ε_s_	27	7	4	2	231	58	26	15
Conduit	ε_e_	57	15	7	4	359	90	40	23
Booster pump	ε_a_	281	71	32	18	496	124	56	32
	**Strain Rate (s** ^**−1**^ **)**								
Reservoir	SR_s_	57	15	7	4	484	121	54	31
Conduit	SR_e_	289	73	33	19	1085	272	121	68
Booster pump	SR_a_	435	109	49	28	869	218	97	55
	**Atrial volume (ml)**								
	Maximum	39	10	5	3	129	33	15	9
	Minimum	85	22	10	6	159	40	18	10
	Prior to atrial contraction	89	23	10	6	147	37	17	10

Presented are sample sizes required to detect a relative 5, 10, 15 or 20 % change in atrial functional parameters (with a 90 % power and an α error of 0.05).

SD, standard deviation; CoV, coefficient of variation; ICC, intraclass correlation coefficient; CI, confidence interval.

## Discussion

The current study aimed to assess the inter-study reproducibility for the analysis of LA and RA dynamics as determined by volumetric indexes as well as CMR-FT derived strain and SR parameters and has several notable findings. Firstly, it shows that the analysis of LA reservoir function is the most reproducible measure using any of the functional indexes (volumetry, strain or SR). Secondly, amongst the different methodology, strain qualifies as the most reproducible parameter for LA functional assessment, whilst in the presence of one anatomical view only, volumetric indexes appear most robust for RA functional assessment. Thirdly, for the first time the performance of CMR-FT for the quantification of RA dynamics alongside its inter-study reproducibility was demonstrated. Lastly, there was no measurable affection of atrial functional parameters by diurnal variation.

Inter-study reproducibility is crucial when repeated examinations are required. As demonstrated previously, CMR-based LV volumetric assessment has high inter-study reproducibility and reduces sample sizes by up to 90 % when compared to echocardiography [17]. It is also important when subtle changes need to be quantified in serial examinations e.g. effects induced by physical exercise or pharmacological interventions. Furthermore, higher inter-study reproducibility bears the potential to improve cost-effectiveness, as fewer subjects are required in clinical trials to detect equal magnitudes of change [18].

CMR-FT represents a relatively novel approach for the quantification of atrial dynamics [8, 10]. Previous CMR-FT studies primarily focused on ventricular strain measurements [19-23]. The reported amount of reproducibility and repeatability varies between studies with most studies reporting reasonable reproducibility of global strain values [8, 19, 21, 23, 24]. As demonstrated previously the highest inter-study reproducibility for LV CMR-FT has been reported for LV global circumferential strain [24]. In contrast, segmental strain parameters were less reproducible [25]. As demonstrated in the present study, the reproducibility of global longitudinal LA strain is as good as reported for global LV circumferential strain [24]. This is most likely a result of averaging strain from all tracked features in the 2- and 4-chamber views based on three analysis repetitions. As opposed to this, CMR-FT derived RA strain and SR profiles were derived from the 4-chamber view only. This most likely explains the inferior inter-study reproducibility of CMR-FT of RA function when compared to LA function analysis. This hypothesis is underlined by the results of the separated analysis of 2- and 4-chamber view derived LA strain and SR, which showed better reproducibility in the 2-chamber view than in the 4-chamber view. LA deformation parameters derived from the 4-chamber view only had similar reproducibility as RA deformation parameters. This is most likely a consequence of the general lower reproducibility of the 4-chamber view, which can be heavily affected by insufficient breath holding as compared to the 2-chamber view, which is less susceptible to diaphragmatic motion. It is important to note that in contrast to CMR-FT, volumetric assessment of atrial function is equally reproducible for the LA and RA. This also might be the result of higher variability of LA CMR-FT in the 4-chamber than in the 2-chamber view, since the circumference of the LA is more often interrupted due to inserting pulmonary veins or the LA appendage in the 4-chamber view. This might negatively impact CMF-FT quality but has no influence on volumetric analysis.

We found better reproducibility for atrial strain than for SR measurements. This is in accordance with previous studies showing inferior inter-study reproducibility of ventricular SR compared with strain [23]. This difference seems to be even more pronounced at 3 T as described by Singh et al. who found worse inter-study reproducibility of LV peak early diastolic circumferential SR at 3 T as compared to 1.5 T [23]. In the present study, all measurements were performed on 3 T, which might explain the inferior reproducibility of SR. Another measure that potentially influences SR more than strain acquisitions is the temporal resolution, which in comparison to speckle tracking echocardiography, is certainly lower for CMR-FT. Unfortunately, there is currently no available data, to define both adequate temporal and spatial resolutions for CMR-FT acquisitions. Future studies will need to define the optimal field strength as well as image resolution for the performance of atrial CMR-FT.

The sample size calculations in the current study demonstrate that relatively small samples are required to detect a 20 % change in LA and RA reservoir function using any technique. Not surprisingly, sample sizes increase with more subtle changes to be detected. It is important to note that the image quality of our healthy volunteers enrolled in the analysis was good to excellent. Reproducibility and sample sizes may therefore be different in patients or when image quality is reduced. However, to partially correct for this, sample sizes calculated in the present study are based on 90 % power, which is higher than commonly performed in patient studies [18, 26]. Furthermore, previous ventricular CMR-FT studies on volunteers at rest and during dobutamine stress demonstrated similar reproducibility irrespective of inotropic stimulation [27], even though dobutamine stimulation may affect image quality. This also holds true for patient studies, which even demonstrated improved CMR-FT reproducibility during dobutamine exposure as compared to measurements at rest in a patient group with coronary artery disease [25]. However, the affection of inotropic stimulation on atrial CMR-FT reproducibility will need to be addressed in future investigations.

### Left atrial dynamics

Although there are data that support the use of LA maximum and minimum volumes for the prediction of mortality in different cardiovascular diseases [28, 29], theoretical considerations and a growing body of literature suggest focussing on the quantification of the three basic atrial functions [2]. Accordingly, more recent CMR investigations increasingly focus on the analysis of LA dynamics using volumetric indexes to quantify atrial reservoir, conduit and contractile booster pump function [2, 4-7]: LA reservoir function has shown to better correlate with LV filling pressures than LA volumes [30] and has demonstrated to be a sensitive biomarker for the prediction of adverse cardiac events independently of other measures of cardiac dysfunction in patients with heart failure [7]. Our study demonstrates excellent inter-study reproducibility for the analysis of LA reservoir function using volumetric indexes, strain and SR parameters. This parameter therefore represents an imaging biomarker, which can be reliably applied in longitudinal studies with repeated measurements.

Strong association between LA conduit function and recurrent atrial fibrillation after pulmonary vein isolation has been described [4]. In the present study, LA conduit function was highly reproducible using volumetric and strain analysis. Moreover, there is evidence that volumetrically quantified impaired LA contractile booster pump has strong prognostic implications for adverse cardiac events in asymptomatic patients at risk for LV diastolic dysfunction [6]. However, our data show considerable inter-study variability for volumetric and SR assessments of LA contractile booster pump function. The better inter-study reproducibility of LA strain quantification of booster pump function certainly needs to be considered when using CMR for this purpose.

Generally, sample size adjustments - as suggested by our data - need to be considered when applying these functional parameters in studies with repeated measurements. The individual merit of the various functional LA elements as determined by the different techniques will need to be addressed in future clinical studies.

### Right atrial dynamics

RA mechanics as assessed by volumetric indexes or deformation parameters have not been studies as much as LA function yet. Most of the present reports focus on RA functional analysis in healthy subjects [13, 31, 32], patients with pulmonary hypertension [33, 34] or congenital heart disease [35]. However, there is more recent evidence that the degree of right ventricular dysfunction is an important prognostic factor in patients with heart failure and preserved ejection fraction [36, 37]. The additional study of RA function, using the proposed methods here, appears highly interesting in this patient group. However, if applied in studies with repeated measurements, the variability of RA functional parameter quantification needs to be addressed. RA reservoir function - as derived from volumetric and strain measurements - has good inter-study reproducibility, whilst there is more variability between repeated measures regarding RA conduit and booster pump function.

### Limitations

The main limitation of the present study is the inclusion of healthy volunteers rather than patients. Reproducibility might vary between healthy volunteers and patients with different cardiovascular disorders. However, previously published data suggest that volunteer and patient reproducibility may well be similar as discussed above. The sample size of this study was relatively small. Ideally, a head - to - head comparison of the inter-study reproducibility between echocardiography and CMR derived atrial phasic functions and deformation parameters would have been performed, as conducted for LV function previously [17].

## Conclusions

The inter-study reproducibility for CMR-based derivation of atrial function is within acceptable limits using either volumetric, strain or SR parameters. With respect to both, LA and RA functional elements, reproducibility is best for reservoir function, followed by conduit and booster pump function. Amongst the different techniques, CMR-FT derived strain qualifies as the most reproducible parameter for LA functional assessment, whilst in the presence of one anatomical view only, atrial volumetric indexes as derived from fractional volume changes appear most robust for RA functional assessment. The degree of inter-study reproducibility of the different methodology and atrial functional elements requires adequate adjustment of sample sizes in future longitudinal studies with repeated measurements.

## Abbreviations

ANOVA: Analysis of variance
CMR: Cardiovascular magnetic resonance
CMR-FT: Cardiovascular magnetic resonance feature tracking
CoV: Coefficient of variation
ε: Strain
EF: Atrial emptying fraction
ICC: Intraclass correlation coefficient
LA: Left atrial
RA: Right atrial
SR: Strain rate, SSFP, steady-state free precession
Vmax: Atrial maximum volume
Vmin: Atrial minimum volume
Vpac: Atrial volume prior to atrial contraction
